# Comparison of edoxaban and enoxaparin in a rat model of AlCl_3_-induced thrombosis of the superior sagittal sinus

**DOI:** 10.1007/s00210-023-02546-x

**Published:** 2023-06-01

**Authors:** M. Hachenberger, M. Yeniguen, L. Suenner, D. Hinchliffe, C. Mueller, A. Wietelmann, T. Gerriets, M. Tschernatsch, M. Juenemann, S. T. Gerner, T. R. Doeppner, H. B. Huttner, T. Braun

**Affiliations:** 1 Department of Neurology, Justus-Liebig University Giessen, Klinikstraße 33, 35385 Giessen, Germany; 2 Heart and Brain Research Group, 35385 Giessen, Germany; 3 NeuroCentrum Wetzlar, Sportparkstrasse 2, 35578 Wetzlar, Germany; 4 Department of Radiology, Kerckhoff-Klinik Bad Nauheim, 61231 Bad Nauheim, Germany; 5 Scientific Service Group Magnetic Resonance Imaging, Max Planck Institute for Heart and Lung Research, 61231 Bad Nauheim, Germany; 6 “Die Neurologen”, Private Practice, Frankfurter Strasse 34, 61231 Bad Nauheim, Germany; 7 Center for Mind, Brain and Behavior (CMBB), University of Marburg and Justus-Liebig-University Giessen, 35032 Marburg, Germany; 8 Department of Neurology, University of Goettingen Medical School, 37075 Goettingen, Germany

**Keywords:** Rat, Sinus thrombosis, Cerebral venous thrombosis, Stroke, Edoxaban

## Abstract

Cerebral sinus venous thrombosis (CSVT) is an uncommon disease that is usually treated with anticoagulation (heparin, low-molecular heparin, or vitamin K-antagonists). We compared treatment with edoxaban, an oral factor Xa-antagonist, that has not been approved in patients with CSVT, with enoxaparin, a well-established therapy, in a rat model of CSVT. Fifty male Wistar rats were randomized into 5 groups (10 animals each) and subjected to aluminum chloride (AlCl3)-induced thrombosis of the superior sagittal sinus (SSS) or sham procedure. Animals with thrombosis of the SSS were treated with edoxaban, enoxaparin, or placebo. Diagnostic workup included neurological examination, MRI imaging, MR-flow measurements of the SSS, and immunohistochemical staining. Neurological examination revealed no differences between treatment groups. Seven days after initial thrombosis, flow in the SSS was lower in the active treatment group as compared to sham-operated animals (*p* < 0.05). Flow in the SSS in the active treatment groups (edoxaban 1 h prior to thrombosis: 0.16 cm/s ± 0.06 cm/s; edoxaban 6 h after thrombosis: 0.13 cm/s ± 0.05 cm/s; enoxaparin: 0.13 cm/s ± 0.04 cm/s; placebo: 0.07 cm/s ± 0.02 cm/s) was higher as compared to placebo (*p* < 0.05), but there were no differences between the active treatment groups (*p* > 0.05). Immunohistochemical staining showed no differences in the actively treated animals. Edoxaban proved to be similar to enoxaparin in a model of experimental AlCl_3_-induced CSVT.

## Introduction

Cerebral sinus venous thrombosis (CSVT) is an uncommon disease and occurs in younger patients. Risk factors are female gender, intake of oral contraceptives or hormone replacement therapy, pregnancy and puerperium, obesity, smoking, and thrombophilia. Symptoms usually develop gradually and consist of headache, focal symptoms, encephalopathy, and epileptic seizures. Treatment consists of anticoagulation (heparin, low-molecular heparin, and vitamin k antagonists (Ferro and Aguiar de Sousa 2019). Recently, the thrombin-inhibitor dabigatran was identified as a safe alternative (Ferro et al. 2021), but has not yet been approved for the therapy of CSVT.

Hence, the present study elucidated alternative state-of-the-art therapeutic options in a preclinical model of CVST. Previous work of our own have already established successful closure of the superior sagittal sinus (SSS) using aluminum chloride (AlCl_3_ (paper in review)). We chose AlCl_3_, because this substance does not lead to artifacts in the magnetic resonance imaging (MRI) as compared to ferric chloride. In the study at hand, we tested the effects of the factor Xa-inhibitor edoxaban in a rat model of AlCl_3_-induced CSVT as compared to the low-molecular-weight heparin enoxaparin.

## Methods

### Animal preparation and experimental design

All animal studies were performed in accordance with institutional guidelines for animal research and were approved by the regional animal care and use committee (Regierungspraesidium Darmstadt, Germany; Az. B10/1000). Fifty male Wistar rats (Charles River, Germany), weighing 276 ± 22.47 g, were used for the present study. The animals had free access to water and food and were kept under conditions of a circadian rhythm. Rats received analgesia with buprenorphine (Buprenovet, Bayer AG, Germany) in a dosage of 0.05 mg/kg body weight subcutaneously (s.c.) half an hour before the anesthetic was administered. The anesthesia was induced with 5% isoflurane (Isofluran CP, CP-Pharma) in 2 l/min oxygen. The maintenance was performed with 2–2.5% isoflurane in 0.5 l/min oxygen. Isoflurane concentrations were controlled during the entire operation within the specified limits, taking physiological parameters into account. The body temperature of each animal was kept constant at 37.0 °C throughout the surgery, using a feedback-heating plate.

SSS thrombosis was induced as previously described (Stolz et al. 2011) with slight modifications. Briefly, as mentioned above, closure of the SSS using ferric chloride, which was used in previous studies, leads to significant artifacts due to its ferromagnetic properties when performing MRI examination, making exact measurements impossible. Therefore we used AlCl_3_ as this substance does not lead to artifacts.

After the operating area had been shaved and aseptically prepared, the local anesthetic lidocaine (lidocaine-HCl 2% injection solution, B. Braun, Germany) was applied s.c. for local anesthesia. A skin incision of about 1.5 cm was made in the midline, and the calotte was exposed. The skull bone was drilled down under water cooling so thinly in the midline along the suture that the SSS shone through the bone lamella. Bregma and lambda sutures served as rostral and caudal boundaries. When lifting and removing the bone lamella, care was taken not to damage the dura mater. A filter paper strip soaked with AlCl_3_ solution (40%) was placed on the exposed SSS for a duration of 5 min. Thereafter, another strip of filter paper was soaked with the corresponding substance and placed on the exposed sinus for 5 min. This procedure was then repeated a second time, resulting in a 3 times 5-min application. The filter paper strip covered the entire exposed SSS.

To ensure a safe thrombosis of the SSS, a time frame of 15 min was chosen, during which the substance could diffuse through the vessel wall. Contact of the surrounding brain tissue with the AlCl_3_ solution was avoided. Animals that received a sham operation were used as controls. The filter paper strip was soaked with sodium chloride solution instead of aluminum chloride. After removing the last strip of filter paper, the surgical field was carefully rinsed with sterile sodium chloride solution. The skin was closed with a continuous suture. The animals were observed until they completely regained consciousness and then returned to their cages.

The animals were treated with the analgesic buprenorphine at the abovementioned concentration on the day of surgery and the first postoperative day. Furthermore, the animals received metamizol via drinking water from 1 day prior to surgery up to and including the fifth postoperative day. Seven days after operation, the animals were decapitated while under deep anesthesia after completing the last MRI, and the brains were collected in formalin for histological analysis.

### Functional assessment

Neurological evaluation was performed prior to anesthesia and 24 h after induction of ischemia. We applied a neurological score with 10 different sensorimotor and coordinative items, as described by Nedelmann et al. (Nedelmann et al. 2007). Furthermore, animals were placed on a rotarod that was continuously accelerated from 0 to 30 rounds per minute (rpm). The maximum speed that the animals tolerated without falling off the device was recorded (Hamm et al. 1994). The rotarod scores before surgery and 24 h or 7 days after surgery were subtracted to display the deterioration.

### MRI studies

MRI measurements were performed using a 7 Tesla MRI spectrometer (PharmaScan, Bruker) equipped with a 760 mT/m gradient system using a 20 mm ^1^H receive-only surface coil together with a 72-mm transmit-only volume resonator. First measurement was performed preoperatively on the healthy animal in order to obtain basic values. Second measurement took place on the first postoperative day and the third measurement on the seventh postoperative day.

During MRI measurements, the anesthesia of rats was induced using 5% isoflurane at 1 l/min oxygen. Subsequently, the animals were fixed in a cradle with a breathing mask, and anesthesia was maintained at 1.5–2% isoflurane at 0.5 l/min oxygen. The cradle was placed into the MRI until the correct positioning was achieved. The rectal temperature of rats was kept at 37.0 °C using a feedback-controlled water bath. The protocol included T1- and T2-mapping sequences, as well as angiography sequences.

After adjustments of field homogeneity, frequency, and transmit amplitudes, localizer scans in three perpendicular directions were acquired. For visualization and volumetric analysis of the volume of edema in the surrounding brain parenchyma where appropriate, a T2-CPMG (Carr Purcell Meiboom Gill) mapping sequence was followed: TR = 3800 ms, NEX = 1, matrix = 512 × 256, FOV = 35 × 35 mm^2^, slice thickness = 1 mm, 12 slices, no gap, TE = 18 to 216 ms in steps of 18 ms. The angiography sequences controlled the degree of occlusion. Due to the low flow velocity in the SSS, the sequences were based on a 3D phase contrast method with an encoding velocity (venc) of 20 cm/s for the detected velocity range: TR = 12 ms, TE = 4,54 ms, NEX = 2; flip angle = 30°, matrix = 256 × 256 × 85, FOV = 30 × 30 × 17 mm^3^, slab thickness = 17 mm. Additionally, a flow map sequence with TR = 15 ms, TE = 4,54 ms, NEX = 8, flip angle = 30°, matrix = 256 × 256 × 11, FOV = 30 × 30 × 11 mm^3^, slab thickness = 11 mm, and venc = 30 cm/s for the flow quantification was included, important particularly in the course of treatment.

### Quantitative image analysis

The calculated images of the different sequences were analyzed with a suitable software program (Paravision 6.0.1 Image Display and Processing, Bruker). Based on the least squares method, T1 and T2 maps were calculated to evaluate possible injury in the surrounding parenchyma and changes of the thrombosis during treatment. A region of interest for flow measurement was defined in the SSS. Based on the angiography sequences, a sagittal image was created showing vessels in which blood flow took place.

### Immunohistochemical staining

The cryofixed rat brain tissue was sliced in 8-μm samples, fixed with 4 °C paraformaldehyde (PFA) 4%, permeabilized by exposure to heated citrate solution and blocked with a bovine serum albumine/glycerine/glycin solution. Primary antibody binding followed by using 10 µg/mL anti-VEGF (vascular endothelial growth factor; vascular tissue, angiogenesis), 1:100 anti-α-SMA (alpha smooth muscle actin; cytosol of vascular smooth-muscle cells), or both, respectively, in a saturated humid chamber for 24 h at 4 °C. Subsequently, the labeling of the anti-VEGF antibody was performed using 1 µg/mL Alexa-488 for 2 h at room temperature. The anti- α-SMA antibody used has been pre-labeled with FITC-514. Counterstaining was performed using a 10 μg/μL DAPI solution. Scanning and analysis of the slides were done with a Zeiss Axio Scan Z1 microscope and the Zeiss ZEN 2.3 lite (blue edition) system. Fluorescence intensities were calculated as relative to the normal brain tissue.

### Randomization and treatment

After baseline-MRI, the animals were randomized to the different groups (10 animals in each group):

1. Edoxaban p.o. (20 mg/kg BW) 1 h before closure of the SSS, afterwards once daily (dosage constant p.o.)
2. Edoxaban p.o. (20 mg/kg BW) 6 h after closure of the SSS, then once daily (dosage constant p.o.)
3. Enoxaparin s.c. (450 IU/kg BW) 6 h after closure of the SSS, followed by twice a day (dosage constant s.c.)
4. Placebo: NaCl p.o. (amount to be administered according to the Edoxaban dosage) 6 h after closure of the SSS, followed by once daily (dosage constant p.o.)
5. Sham operation: no treatment

Edoxaban and placebo were administered via gavage. The persons performing and analyzing the MRI examination or the immunohistological examination were blinded. Edoxaban was supplied by Daiichi Sankyo Co., Ltd. (Tokyo, Japan).

### Inclusion and exclusion criteria

Animals were included if the MRI showed a sufficient reduction of the flow in the SSS. Prespecified exclusion criteria were unstoppable bleeding during surgery (e.g., laceration of the SSS) or model failure. The animals were also excluded in the case of severe dyspnea and neurological impairment that made the ingestion of food and water impossible.

### Outcome measures

Neuroscore, flow in the SSS (primary outcome), presence of parenchymal damage, and immunohistochemical changes 7 days after induction of thrombosis were defined as outcome measures.

### Data evaluation and statistical analysis

All data are given as mean ± standard deviation (SD). Data were tested for normal distribution and variance homogeneity. The Kruskal–Wallis test was used to test the functional assessments. A *t* test for independent samples was then performed. The values of the 2 time points (preoperative, 7 days postoperative) were compared, and *p* < 0.05 was considered statistically significant. The data analysis program SPSS (IBM) was used for evaluation. The sample size was decided due to observations from previous work when developing the experimental method (paper in review).

## Results

Only 30 animals (75%) completed the experiment and were analyzed per protocol. Four animals died during the surgery and MRI examination procedure (10%) due to respiratory failure. The MRI examination detected model failure (SSS not closed) in 6 (15%) animals. For the distribution of the surviving animals, see Table 1.

**Table 1:** Distribution of surviving animals in the different treatment groups

Group	Number of surviving animals
1: Edoxaban 1 h prior to SSS closure	5
2: Edoxaban 6 h after SSS closure	7
3: Enoxaparin 6 h after SSS closure	7
4: Placebo	5
5: Sham operation	6

### Functional evaluations

Seven days after closure of the SSS, clinical evaluation using the neuroscore revealed no differences between groups (*p* > 0.05). Rotarod testing also showed no differences between the groups (*p* > 0.05).

### MRI examinations

**Fig. 1:**
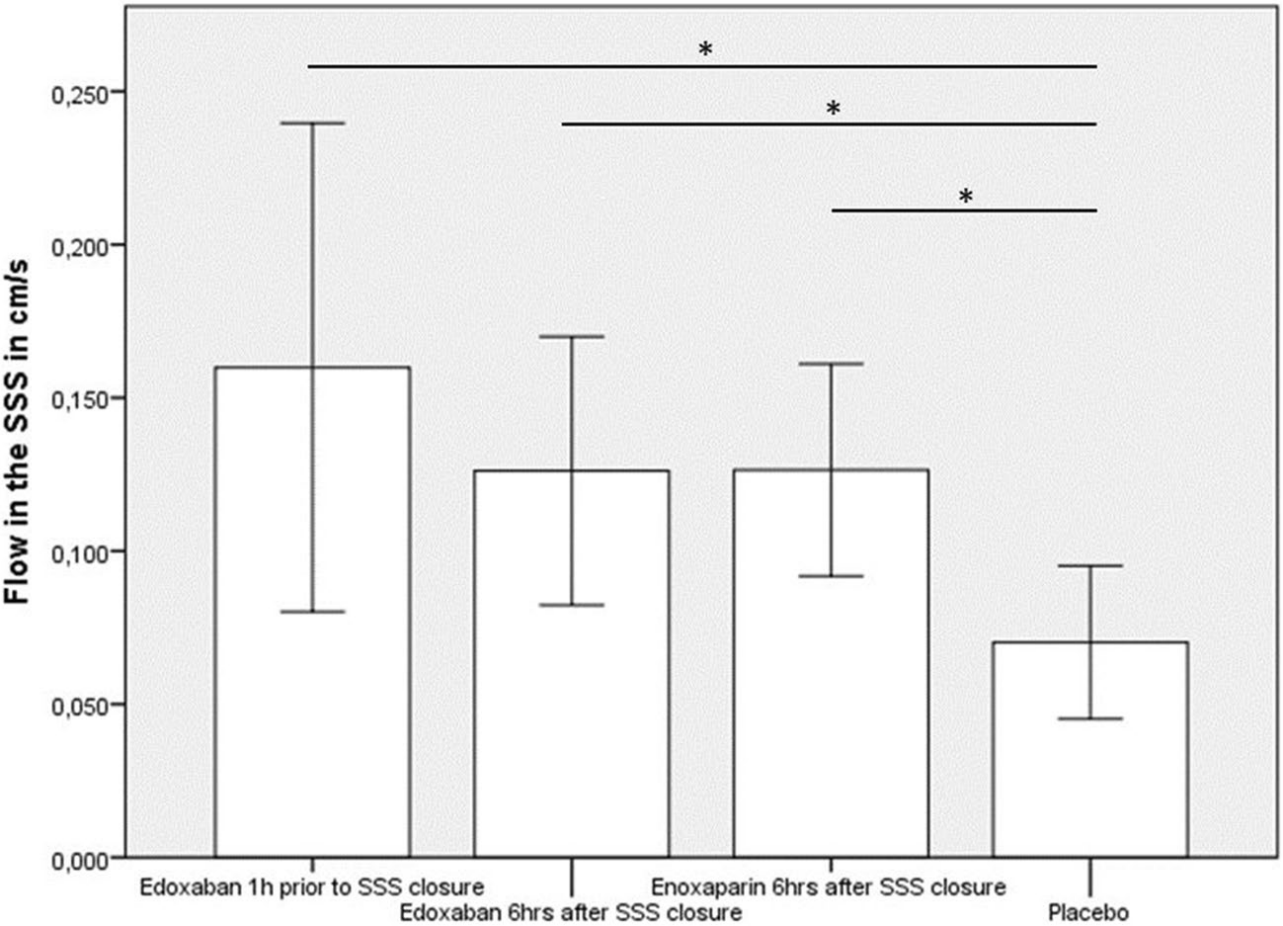
Flow in the superior sagittal sinus in cm/s. The data is presented as means and standard deviation. Asterisk marks a *p*-level < 0.05

Flow measurements showed significant reduction in groups 1–4 as compared to sham (*p* < 0.05), indicating a successful closure of the SSS. All active treatment groups showed a better flow in the SSS as compared to placebo (group 1: 0.16 cm/s ± 0.06 cm/s; group 2: 0.13 cm/s ± 0.05 cm/s; group 3: 0.13 cm/s ± 0.04 cm/s; group 4: 0.07 cm/s ± 0.02 cm/s; *p* < 0.05). However, there were no differences between the active treatment groups (*p* > 0.05) (Fig. 1). We detected no parenchymal damage in the animals 7 days after closure of the SSS.

### Immunohistochemical staining

We detected no differences in the fluorescence intensities between the treatment groups for VEGF (group 1: 0.243 ± 0.344; group 2: 0.075 ± 0.121; group 3: 0.042 ± 0.067; group 4: 0.017 ± 0.049; group 5: 0.058 ± 0.075; *p* < 0.05) or for alpha-SMA (group 1: 0.075 ± 0.05; group 2: 0.1145 ± 0.132; group 3: 0.091 ± 0.064; group 4: 0.057 ± 0.034; group 5: 0.103 ± 0.082; *p* < 0.05).

## Discussion

In our AlCl_3_-mediated model of CSVT in rats, both edoxaban and enoxaparin showed a significant increase of SSS flow. Of note, a direct head-to-head comparison in a preclinical model of CSVT has not yet been performed, providing valuable data with a clinical impact. Edoxaban is a fast-acting anticoagulant that has been approved for treatment of non-valvular atrial fibrillation as well as prevention and treatment of venous thrombosis. The mechanism of action is a direct inhibition of the factor Xa (Hurst et al. 2016). In an in vitro study on human clots, edoxaban inhibited tissue factor-induced platelet aggregation in a concentration-dependent manner (Honda et al. 2016). Interestingly, another way of action where edoxaban acts in a plasmin-independent profibrinolytic effect is also suggested. Such data is backed up due to the finding of thinner fibrin fibers and larger pores in clots formed in the presence of edoxaban compared with control clots (Morishima et al. 2020).

In rodents, edoxaban was found to effectively prevent thrombosis non-related to CSVT (Furugohri et al. 2008). In an lipopolysaccharide model of microvascular thrombosis in rats, edoxaban inhibited the hypercoagulation and fibrin deposits in the liver and reduced mortality following lipopolysaccharide injection (Morishima et al. 2021). In a platin wire model of thrombosis of the inferior vena cava in rats, edoxaban showed effects similar to enoxaparin in regards to the thrombosis. The bleeding risk was, however, lower in the edoxaban group (Morishima et al. 2012). Under conditions of ferric chloride-induced venous thrombosis, edoxaban leads to upregulation of hydrogen sulfide and homocysteine activities through the MMP-9-induced PI3K/AKT signaling pathway (Song et al. 2017). This pathway is thought to inhibit the activation of platelets in thrombus formation and stabilization (Guidetti et al. 2015).

When CSVT is suspected in humans, the diagnosis is confirmed by venous angiography with contrast-enhanced CT or MRI. Intracerebral hemorrhages as a result of venous infarction can be detected as well. After confirmation of the diagnosis, patients are started on an anticoagulative regime, irrespective of the presence of intracerebral hemorrhages. The treatment usually consists of unfractioned heparin or low-molecular heparin in full anticoagulant dosage followed by an administration of vitamin K-antagonists for 3 to 12 months (Ferro and Aguiar de Sousa 2019). Venous recanalization is achieved in 85% of patients, but there is only limited data on the temporal profile (Kenet et al. 2007).

Recently, the RE-SPECT-CVT study showed that treatment with dabigatran may be safe and effective in treating CSVT. However, dabigatran has not yet been approved for the treatment of CSVT (Ferro et al. 2021). Edoxaban might have a similar profile of safety and efficacy in the treatment of CSVT, but edoxaban offers the advantage of a single daily dose only. In light of edoxaban being as effective as enoxaparin, edoxaban might be a useful alternative to dabigatran and vitamin k antagonist in long-term treatment paradigms of patients suffering from CVST.

The main limitation of our experiment, however, is the short observation period of 7 days only. One might argue that the time is not sufficient to detect side effects such as bleeding events and that a recanalization is unlikely to occur in 7 days. Nevertheless, we were able to detect a higher flow in the SSS of rats treated with either edoxaban or enoxaparin, indicating an already beginning, albeit putative, recanalization of the formerly occluded vessel. Beside the limited observational time window of our study, the surgery technique itself as well as the way of inducing an SSS should also be considered. Since the composition and structure of the thrombus produced by AlCl_3_ cannot be determined, no statement can be made about the consistency and integrity of the thrombus. It is possible that the drugs studied herein are less effective because the thrombus is too well organized. This would not correspond to the thrombus characteristics after SSS in humans.

## Conclusion

Using a well-established preclinical model of CSVT, treatment with edoxaban proves to be similar to the standard of care treatment paradigm administering enoxaparin. Hence, edoxaban might offer an alternative and feasible treatment paradigm for patients suffering from CSVT.

## Data Availability

The datasets used and/or analyzed during the current study are available from the corresponding author on reasonable request.

## Abbreviations

AlCl_3_: Aluminum chloride
CSVT: Cerebral sinus venous thrombosis
MRI: Magnetic resonance imaging
SSS: Superior sagittal sinus
